# A knowledge graph for crop diseases and pests in China

**DOI:** 10.1038/s41597-025-04492-0

**Published:** 2025-02-06

**Authors:** Rongen Yan, Ping An, Xianghao Meng, Yakun Li, Dongmei Li, Fu Xu, Depeng Dang

**Affiliations:** 1 School of Information Science and Technology, Beijing Forestry University, Beijing, 100083 China; 2 Engineering Research Center for Forestry-oriented Intelligent Information Processing of National Forestry and Grassland Administration, Beijing, 100083 China; 3 School of Artificial Intelligence, Beijing Normal University, Beijing, 100875 China

**Keywords:** Agroecology, Forest ecology

## Abstract

A standardized representation and sharing of crop disease and pest data is crucial for enhancing crop yields, especially in China, which features vast cultivation areas and complex agricultural ecosystems. A knowledge graph for crop diseases and pests, acting as a repository of entities and relationships, is crucial conceptually for achieving unified data management. However, there is currently a lack of knowledge graphs specifically designed for this field. In this paper, we propose CropDP-KG, a knowledge graph for crop diseases and pests in China, which leverages natural language processing techniques to analyze data from the Chinese crop diseases and pests image-text database. CropDP-KG covers relevant information on crop diseases and pests in China, featuring 8 primary entities such as diseases, symptoms, and crops, and is organized into 7 relationships such as primary occurrence locations, affected parts and suitable temperature. In total, it includes 13,840 entities and 21,961 relationships. In the case studies presented in this research, we also show a versatile application of CropDP, namely a knowledge service system, and have released its codebase under an open-source license. The content of this paper provides a guide for users to build their own knowledge graphs, aiming to help them effectively reuse and extend the knowledge graphs they create.

## Background & Summary

Artificial intelligence (AI) has been demonstrated to play a contributory role in mitigating agricultural losses, bolstering the sustainability of agricultural production, and propelling the advancement of modern agriculture^1–3^. Each year, crop diseases and pests cause over $70 billion in economic losses worldwide, severely impacting crop yields^4^. Knowledge on crop diseases and pests, collected and effectively analyzed using AI technology, have become a crucial basis for improving crop yields^5–8^. With the advancement of computer technologies, many cloud systems have been established alongside scientific literature repositories, providing convenient ways to store, manage, and share large amounts of data. Examples of such systems include Anhui Province Integrated Crop Diseases and Pests Management Platform (https://www.agrowalker.com/), Tortricid.net (http://www.tortricidae.com/catalogue.asp), FAO’s Agricultural Stress Index System (https://asis.apps.fao.org/), PestNet (https://app.pestnet.org/), Integrated Pest Management Centers (https://www.gpdd.info/) and Plantwise (https://www.cabi.org/plantwiseplus/impact/plantwise/). These areas of knowledge encompass topics such as crop distribution and growth patterns, the biological characteristics of pests, and related visual documentation. However, this knowledge is often scattered across databases, scientific literature, web pages, and various sub-platforms, which limits comprehensive exploration and presents challenges in integrating it into a unified representation.

The constraints on information dissemination and the state of unstructured data significantly impede the innovation and execution of diseases and pests control strategies^9^. This phenomenon of isolated information and inadequate automation has rendered research in crop diseases and pests control relatively stagnant compared to other cutting-edge fields such as agricultural landscape design^10^ and precision farming^11^. Researchers are frequently compelled to rely on the expertise of domain specialists and the existing literature to navigate the challenges of querying data on crop diseases and pests, including the precise identification of specific diseases and pests characteristics, a systematic enumeration of crop varieties with particular resistance genes, the correlation of specific control methods with diseases and pests, and the verification of the implementation of specific control strategies. This process is not only laborious and time-consuming but also susceptible to biases due to incomplete or outdated information. Given this, establishing a unified and structured knowledge representation of crop diseases and pests will not only standardize the identification of relevant knowledge but also integrate dispersed data resources, significantly enhancing the accuracy and efficiency of information retrieval. This initiative will promote interdisciplinary and cross-domain data integration, providing agricultural research with richer and deeper insights, thereby advancing agricultural science to higher levels. It will empower researchers with the capability to swiftly and accurately access critical information, enabling them to effectively tackle challenges posed by diseases and pests, and strengthening collaboration between agricultural researchers and communities. Therefore, the construction of a unified and structured representation of crop diseases and pests knowledge appears particularly urgent.

Knowledge graph is a crucial method for addressing these challenges, embodying a structured approach to representing knowledge based on semantic relationships, aimed at capturing and expressing inter-entity relations^12–14^. Utilizing a graph-based data structure, it organizes information into nodes, representing entities, and edges, symbolizing relationships, thereby facilitating a deeper understanding of data interdependencies and intricacies^15^. Within knowledge graphs, entities and relationships are articulated through standardized semantic labels, enabling interoperability and integration across diverse data sources^16^. For example, Google’s knowledge graph systematically gathers and showcases a plethora of global entities, including individuals, locales, organizations, and events in a structured manner, allowing users to delve more comprehensively into related themes via search engines^17^. Due to its highly structured nature, knowledge graphs have applications across diverse fields such as materials science^18,19^, education^20,21^, medicine^14,22,23^, life sciences^24^, and pharmaceuticals^25^. In the field of agriculture, there are already several knowledge graphs aimed at integrating and standardizing the existing forms of knowledge storage and representation in the agricultural domain. Qin *et al*.^26^ proposed a dual-mode intelligent construction method for agricultural knowledge graphs, which includes key technologies of entity relationship joint modeling. This method focuses on extracting irregular data and integrating entity knowledge through similarity calculation to enhance the standardization, accuracy, and completeness of the graph. Zhu *et al*.^27^ constructed a pest and disease knowledge graph specifically for lychee and longan. Gao *et al*.^28^ designed and implemented an intelligent detection model based on deep learning, and established a knowledge graph of cotton pests and diseases for rapid detection. However, current research and applications of agricultural knowledge graphs predominantly focus on single crops, resulting in relatively limited coverage of entities and relationships. Moreover, the construction of these graphs heavily relies on labor-intensive manual processes, increasing the likelihood of errors and omissions. This approach also extends the construction cycle and diminishes overall efficiency. Consequently, there is an urgent need to develop more automated and intelligent methods to enhance the accuracy and efficiency of knowledge graph construction in this domain.

Natural language processing (NLP), as a pivotal branch of AI, focuses on endowing computers with the capability to parse, comprehend, and generate human language. In the complex task of constructing knowledge graphs, NLP plays a central role. Leveraging state-of-the-art algorithms and models, NLP technology accurately identifies named entity recognition (NER) in text, such as recognizing names of people, geographical locations, organizations, and annotates these entities precisely. Furthermore, NLP excels in relation extraction (RE), revealing semantic connections like identifying “apple” as a type of “fruit”. Moreover, NLP technology encompasses a broad spectrum of information extraction (IE) fields in the process of constructing knowledge graphs. The application of deep learning models significantly enhances NLP’s ability to handle subtle nuances in language, enabling deeper contextual understanding and capturing the underlying semantics of language. The introduction of these technologies not only accelerates the pace of knowledge graph construction but also markedly improves the precision and efficiency of automated construction processes. In our previous research^29^, we successfully developed an automated method capable of accurately extracting key data from scientific literature, laying a solid foundation for our subsequent studies. Nevertheless, the dispersed nature of numerous data items across multiple databases continues to pose a highly challenging task for effective knowledge retrieval and integration, underscoring its inherent complexity.

In this paper, we introduce CropDP-KG, a knowledge graph specifically designed for crop diseases and pests data. The data is sourced from relevant textual materials in the Chinese crop diseases and pests image-text database (http://bcch.ahnw.cn/). This database compiles rich information including categories, types, labels, detailed descriptions of pests and diseases, and images, with detailed disease and pest information primarily in unstructured text format. By applying NLP, we successfully automated the construction of CropDP-KG from these texts. CropDP-KG encompasses a range of key entities such as Chinese Name for Diseases and Pests, English Name and Binomial Nomenclature for Diseases and Pests, Symptoms, Crops Name, Occurrence Conditions, Affected Parts, Occurrence Regions, and Optimal Temperature. It defines relationships among these entities, such as “RegionIs”, “EnglishNameIs”, “ConditionIs”. CropDP-KG, with 13,840 entities and 21,961 relationships, enriches agricultural pest and disease data resources while offering a valuable information platform for research and practical applications in agriculture. To enhance the application of the constructed knowledge graph, we have meticulously designed and implemented a crop diseases and pests knowledge service system. This system integrates functionalities such as knowledge querying, overview, question answering, and management. Supported by CropDP-KG, the platform provides users with a diverse interface for knowledge retrieval, significantly enriching channels for accessing specialized knowledge and offering users more comprehensive and extensive information resources. Through this system, our goal is to advance the dissemination and application of agricultural pest and disease knowledge, providing robust information support for agricultural research and practice.

## Methods

### Text acquisition

We obtained textual data as raw data from Chinese crop diseases and pests image-text database. This database is an independent development by the Institute of Information Research, Anhui Academy of Agricultural Sciences. Over a long period of systematic information collection and in-depth research, the institute has meticulously organized and integrated a large amount of detailed primary data, covering various forms including images, text, and videos. Currently, the database has collected information on more than 5,000 common crop pests in Chinese agricultural production, each entry accompanied by carefully selected high-quality images, totaling 7,034 images. It ranks among the largest databases of its kind in China. The establishment of this database not only provides robust technical support for plant protection issues in Chinese agricultural production but also greatly promotes the scientific management and effective control of agricultural pests, making significant contributions to the sustainable development of Chinese agriculture. We employed web scraping technology to efficiently gather comprehensive data on 3,493 types of crop diseases and pests. The data includes crucial information such as the Chinese and English names, descriptions, major symptoms, occurrence cycles, and has been meticulously organized and stored in CSV files for further analysis and application.

We employ Python web scraping techniques to efficiently gather information from the Chinese crop diseases and pests image-text database. This dataset includes both semi-structured and unstructured data types. For semi-structured data, we develop customized parsing algorithms specifically designed to extract key attributes such as names, English names, aliases, and other critical information from HTML tables and JSON-formatted data. For unstructured textual data, including detailed descriptions, symptoms, and causative factors of agricultural pests and diseases, our approach involved initial data cleaning to remove non-informative content like HTML tags and special characters. Subsequently, employing a series of predefined entity categories, we conducted in-depth processing and systematic classification of the text data to ensure accuracy and applicability in our research.

### CropDP-KG data extraction

**Fig. 1:**
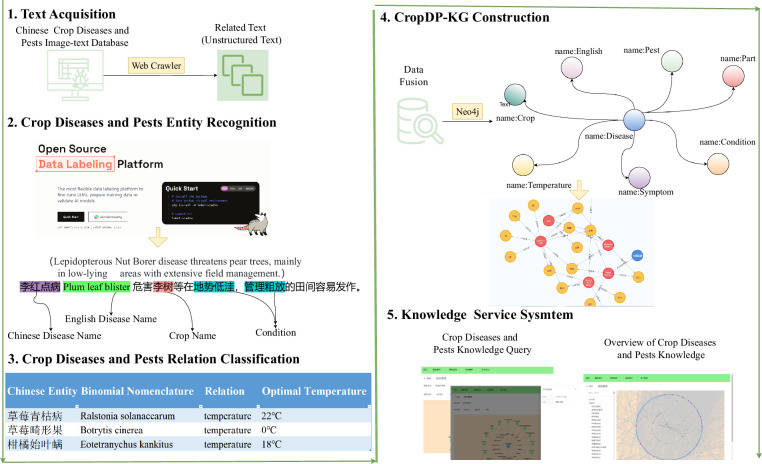
The construction process of CropDP-KG and its application within the system.

The complete construction process of CropDP-KG is depicted in Fig. 1, detailing the establishment of CropDP-KG, encompassing key steps and its applications. CropDP-KG is a knowledge graph dedicated to agricultural pest information, considering data as its core driving force and extensively mining valuable insights from vast agricultural datasets. We meticulously select and analyze critical textual data across multiple dimensions such as crop types, planting regions, and growth cycles. We employ NLP techniques including NER and RE to achieve precise data extraction and deep integration. Through these steps, CropDP-KG successfully constructs a content-rich and structurally clear agricultural knowledge graph, providing robust knowledge support and data-driven decision-making foundations for research and practice in the agricultural domain. Furthermore, leveraging CropDP-KG, we have developed an integrated platform that combines multiple functionalities including knowledge querying, question answering, and knowledge management. This unified system design not only significantly enhances the practical value of crop diseases and pests knowledge but also greatly improves user convenience, making the acquisition and application of agricultural expertise more efficient and intuitive.

**Fig. 2:**
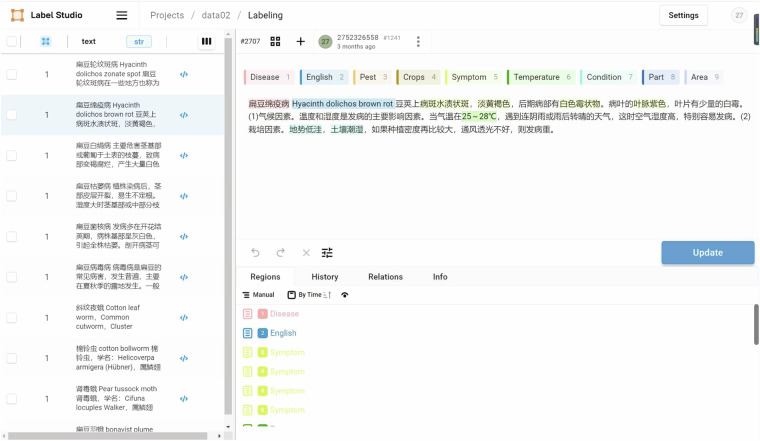
Named entity recognition annotation interface using Label Studio.

In our study, for the precise identification and classification of entities related to crop diseases and pests, we utilized Label Studio, an advanced data annotation tool, to meticulously annotate a large corpus of sentences, as shown in Figure 2. Label Studio is a powerful text annotation platform that supports user-defined entity categories and facilitates efficient data annotation through an intuitive graphical user interface. It as a versatile data annotation platform, excels in creating custom labeling interfaces suitable for various data types such as text, images, audio, video, and time series. The tool’s flexibility and scalability make it indispensable for deep learning tasks. Through Label Studio, we successfully annotated eight key entity categories including Chinese and English names for diseases and pests, crop names, occurrence conditions, affected parts, occurrence regions, symptoms, and optimal temperatures. Considering that the English names of crop diseases and pests fall under structured data, we defined six entity categories other than English names in our unstructured data. We utilized the BMES (Beginning, Middle, End, Single) annotation method to clearly delineate different parts of entities, as detailed in Table 1. Leveraging Label Studio’s robust capabilities, we completed a total of 13,840 high-quality annotations of entities. These annotations were carefully curated by domain experts using Label Studio, ensuring their accuracy and providing a solid foundation for subsequent data-driven research endeavors. To reduce future reliance on expert intervention, we train a dedicated named entity recognition model for crop diseases and pests based on entities annotated from 1500 sentences.

**Table 1:** The annotation format of six entities in unstructured data.

Label name	Label meaning
B-Disease/M-Disease/E-Disease	Disease head word / Disease middle word / Disease tail word
B-Crops/M-Crops/E-Crops	Crop head word / Crop middle word / Crop tail word
B-Symptom/M-Symptom/E-Symptom	Symptoms head / Symptom middle word / Symptom tail word
B-Condition/M-Condition/E-Condition	Conditions head word / Condition middle word / Condition tail word
B-Part/M-Part/E-Part	Part head word / Part middle word / Part tail word
B-Area/M-Area/E-Area	District head word / District middle word / District tail word
O	Non-entity word

**Fig. 3:**
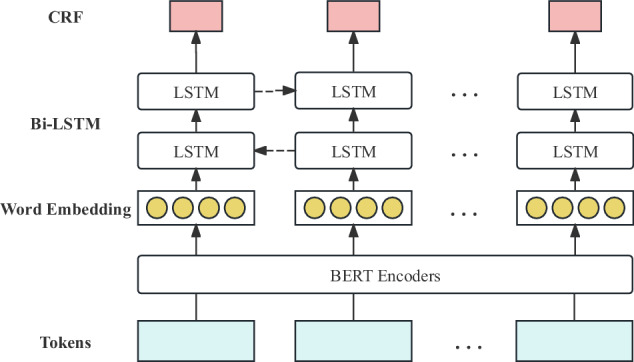
BERT-BiLSTM-CRF model architecture.

For the task of identifying crop diseases and pests entities, we employed two advanced model architectures: one based on BERT with bidirectional long short-term memory networks and conditional random fields^30,31^ (BERT-BiLSTM-CRF), and another simplified model combining BERT with conditional random fields^32^ (BERT-CRF). Both approaches leverage deep learning techniques to enhance the accuracy and efficiency of named entity recognition. Figure 3 illustrates the architecture of the BERT-BiLSTM-CRF model. BERT^33^, as a cutting-edge pre-trained language model, leverages its bidirectional Transformer architecture to deeply capture the semantic information of words within complex contexts. The BiLSTM^34^ module focuses on capturing long-range dependencies in sequential data by processing both forward and backward information flows, enhancing the understanding of context and allowing for more accurate recognition of word relationships. CRF^35^, a sequence labeling model, improves the quality of label sequence predictions by modeling dependencies between labels. The BERT-CRF model simplifies the BiLSTM component by cleverly integrating the rich contextual information provided by BERT with the sequence optimization capabilities of CRF.

The performance of the two models in identifying various types of entities has been quantitatively evaluated and summarized in Table 2. In the table, “P” represents Precision, “R” represents Recall, and the *F*_1_ score serves as a comprehensive evaluation metric. The calculation formula for *F*_1_ score is shown below.

1 $${F}_{1}=2\times \frac{\,{\rm{Precision}}\times {\rm{Recall}}}{{\rm{Precision}}+{\rm{Recall}}}$$

**Table 2:** The named entity recognition results of different model.

Entity Name	BERT-CRF			BERT-BiLSTM-CRF		
	P	R	*F*_1_	P	R	*F*_1_
Disease& Pest	99.40%	96.45%	97.89%	99.21%	96.46%	94.47%
Crop	93.28%	93.42%	93.29%	94.42%	95.82%	95.10%
Symptom	80.10%	92.63%	85.89%	90.52%	93.48%	91.97%
Condition	74.43%	92.40%	82.44%	90.52%	87.32%	86.61%
Part	97.53%	71.72%	77.12%	97.99%	98.51%	98.25%
Region	59.28%	65.76%	62.35%	61.80%	66.48%	64.05%
O	99.23%	97.53%	98.37%	99.42%	98.82%	99.12%
Average	86.18%	87.13%	85.34%	89.90%	90.98%	89.94%

This formula reflects the balance of the model’s recognition capability. Precision measures the proportion of correctly predicted positive instances out of all instances predicted as positive by the model, while Recall measures the proportion of correctly predicted positive instances out of all actual positive instances. A higher *F*_1_ score indicates that the model achieves a better balance between Precision and Recall, thereby performing better in entity recognition tasks.

The table 2 summarizes the performance of two named entity recognition models: BERT-CRF and BERT-BiLSTM-CRF. The BERT-BiLSTM-CRF model consistently demonstrates superior performance in most entity categories compared to BERT-CRF. Notably, BERT-BiLSTM-CRF achieves higher *F*_1_ scores for entities such as “Crop” (95.10% vs. 93.29%) and “Symptom” (91.97% vs. 85.89%). Despite BERT-CRF’s better *F*_1_ score in the “Disease & Pest” category (97.89% vs. 94.47%), the BERT-BiLSTM-CRF model yields an overall higher average *F*_1_ score of 89.94%, compared to 85.34% for BERT-CRF. This suggests that the BERT-BiLSTM-CRF model offers improved accuracy and robustness across various named entity types.

To further construct an agricultural pest and disease knowledge graph, beyond accurate entity recognition, it is crucial to uncover inherent relationships between entities by constructing triplets. In this process, we enlisted numerous domain experts who meticulously extracted 21,961 triplets from annotated texts leveraging their profound expertise. These triplets encompass seven primary relationship types, such as <pest/disease name, “RegionIs”, occurrence region>, <pest/disease name, “ConditionIs”, occurrence condition>, <pest/disease name, “Damage”, crop name>, <pest/disease name, “EnglishName”, full English name>, <pest/disease name, “InflictHarmPart”, affected part>, <pest/disease name, “ManifestAs”, symptoms>, and <pest/disease name, “TemperIs”, Optimal Temperature>. These carefully curated triplets not only deepen our understanding of pest and disease interrelations but also significantly expand the depth and breadth of the knowledge graph, providing rich data resources for agricultural scientific research and practical applications. Furthermore, these triplets serve as a foundational basis for future model construction, despite this study not directly involving training of relationship extraction models. The aim of this paper is to establish a solid data foundation for subsequent in-depth research, paving the way for further development and application possibilities in agricultural knowledge graphs.

### CropDP-KG construction and visualization

Building upon successful NER, we proceeded with RE. Through careful curation by experts, identified entities were transformed into knowledge triplets. These triplets not only capture direct connections between entities but also delve deeper into the complex interactions among entities in the field of crop diseases and pests. One key step in the construction of CropDP-KG is to effectively integrate the entity and relationship triplets related to crop diseases and pests into a knowledge graph.

To achieve this, we adopted Neo4j, a high-performance graph database known for its fast processing capabilities, unlimited scalability, security, and data integrity. Neo4j is particularly suitable for task-critical intelligent applications. Unlike traditional relational databases, Neo4j stores nodes and relationships instead of tables or documents, implementing a true graph model even at the storage level. Neo4j, as a leading tool for automated knowledge graph construction, excels in its core advantage of automation, providing users with an intuitive graphical thinking model to organize and gain insights from data. This significantly enhances both the depth and breadth of data comprehension. Its Cypher query language, known for its concise yet powerful syntax, further simplifies the creation and querying of data relationships, facilitating automated knowledge graph construction and user-friendliness. This automation not only reduces the complexity of manual operations but also improves efficiency in building knowledge graphs, making the process more effective, intuitive, and manageable.

**Fig. 4:**
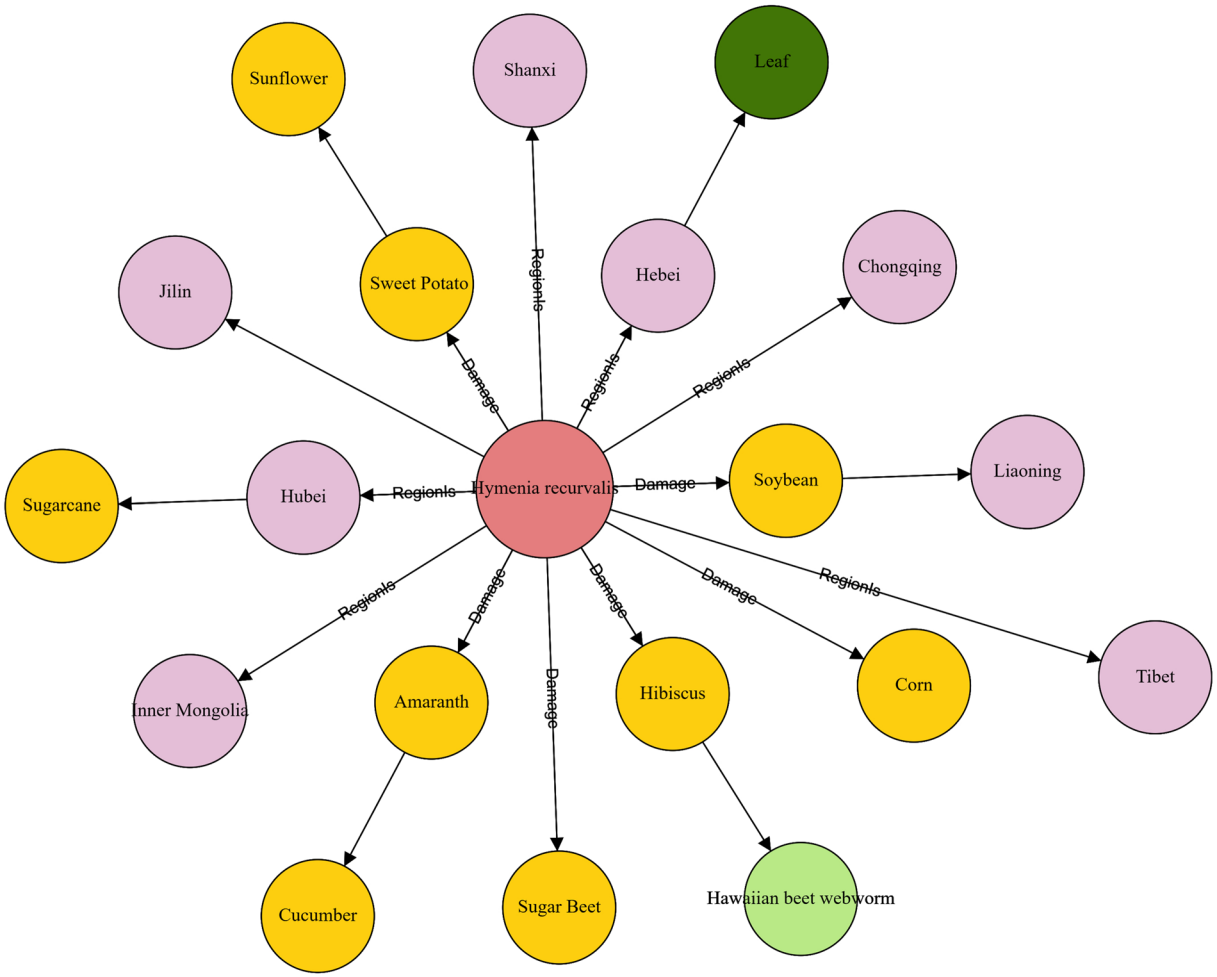
Visualization example of crop diseases and pests knowledge graph.

Figure 4 depicts a partial example of the knowledge graph generated after importing crop disease and pest data into Neo4j. The nodes in the figure represent different entities, encompassing seven distinct types, each distinguished by a unique color. The figure also illustrates four fundamental types of relationships: “RegionIs”, “Damage”, “EnglishName” and “InflictHarmPart”. Additionally, our constructed knowledge graph also includes three crucial relationships termed ‘TemperIS’, ‘ConditionIs’ and ‘ManifestAs’, although these are not explicitly shown in the current visual example. “RegionIs” indicates the geographical region or distribution range where a pest or disease typically occurs. “ConditionIs” describes the specific conditions or environmental requirements for the occurrence of the pest or disease. “Damage” specifies the impact or harm that the pest or disease causes to specific crops. “EnglishName” provides the complete English name of the pest or disease. “InflictHarmPart” details the specific parts of plants or crops affected by the pest or disease, such as leaves, roots, or fruits. “ManifestAs” describes the typical symptoms or manifestations of the pest or disease, such as yellowing leaves, rotting fruits, or weakened plants. “TemperIs” indicates the optimal temperature range for the occurrence and reproduction of the pest or disease. These relationships form the foundational structure of the agricultural pest and disease knowledge graph, facilitating a comprehensive understanding of their distribution, characteristics, and influencing factors.

### Knowledge service system

To facilitate users in fully leveraging our developed CropDP-KG, we create a web system - the Crop Diseases and Pests Knowledge Service System. This system aims to provide comprehensive information on crop diseases and pests. Utilizing the popular frontend-backend separation architecture, it not only enhances system performance but also greatly improves maintainability. The platform’s implementation code is publicly available on GitHub (https://github.com/dadadaray/CropDP-KG/tree/Knowledge-System) to promote knowledge sharing and technological exchange.

The backend of the platform is developed using Python and the Django framework (https://www.djangoproject.com/), ensuring efficient and stable data processing. For the frontend, Vue framework (https://v2.nuxt.com/) is employed alongside HTML, CSS, and other technologies, carefully crafting a user interface that combines aesthetics with practicality. We have also enriched the interface using the Element-UI component library, enhancing the user experience with modern design principles. In terms of knowledge graph visualization, we integrated the cytoscape.js library within Vue to intuitively present the knowledge structure graphically to users, enabling interactive operations through interfaces. Data interaction between frontend and backend is efficiently handled using the Axios (http://www.axios-js.com/) asynchronous request library, ensuring responsive system performance and smooth user interactions.

**Fig. 5:**
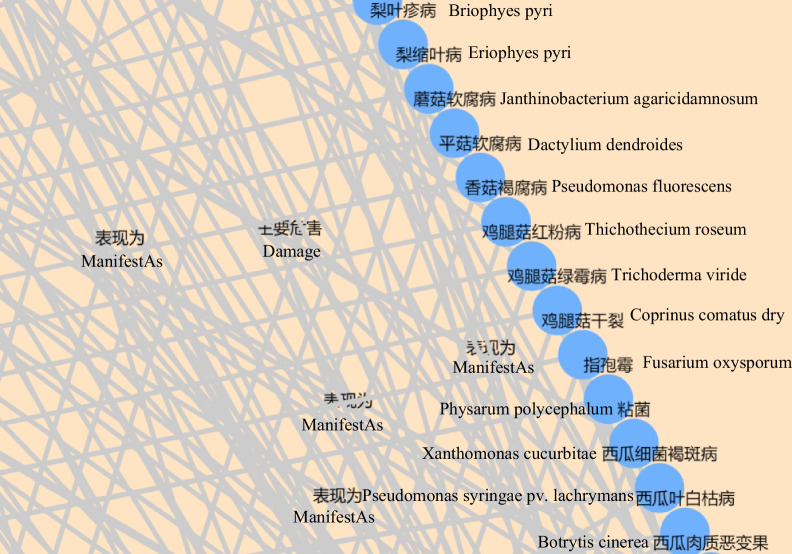
The detailed information on crop diseases and pests, with examples visible upon mouse zoom-in.

Functionalities of the system include knowledge graph display, querying capabilities, and system maintenance tools, aiming to provide users with a comprehensive, efficient, and user-friendly platform for crop diseases and pests knowledge services. Knowledge overview page of the Crop Diseases and Pests Knowledge Service System built upon CropDP-KG. On the left side of the page, a comprehensive list of all crops and their associated pest and disease information is detailed, providing users with convenient access for quick retrieval and browsing. The right side of the page features an intuitive overview of the entire knowledge graph, graphically displaying the intricate relationships between crops, diseases and pests. The view on the right supports zoom in and zoom out operations, enabling users to flexibly examine either detailed aspects or the overall structure of the knowledge graph as needed, thereby facilitating a richer and deeper knowledge experience. Figure 5 further elaborates on a zoomed-in local view of the knowledge graph. Through this zoomed-in perspective, users can clearly observe specific entities and their detailed relationships.

The creation of CropDP-KG has brought revolutionary advancements to the agricultural sector. Leveraging an intuitive graphical interface, it significantly enhances users’ capability to access and comprehend information on crop diseases and pests. In terms of knowledge querying, users can swiftly locate detailed information on specific crops or pests, gaining insights into their interactions and influencing factors, thus providing robust data support for agricultural decision-making. Furthermore, CropDP-KG’s knowledge question-answering feature utilizes intelligent analysis to accurately respond to user queries, effectively addressing practical issues. This question-answering not only improves decision-making efficiency but also enhances accuracy, providing strong assistance for agricultural management. Moreover, the knowledge management functionality ensures the timeliness and reliability of information within the knowledge graph through systematic and automated maintenance and updating mechanisms. This not only reduces the burden of manual maintenance but also guarantees the continuous updating and accuracy of the knowledge graph. The applications of CropDP-KG extend beyond these capabilities. It serves as a powerful tool for researchers, agricultural workers, and policymakers, supporting their work in disease and pest control, crop health management, and sustainable agricultural development. Through CropDP-KG, users can efficiently leverage knowledge resources to optimize agricultural practices, promoting the health of agricultural ecosystems. In conclusion, the construction of CropDP-KG represents not only technological progress but also a crucial step towards enhancing knowledge management and decision intelligence in agriculture.

#### Knowledge querying

**Fig. 6:**
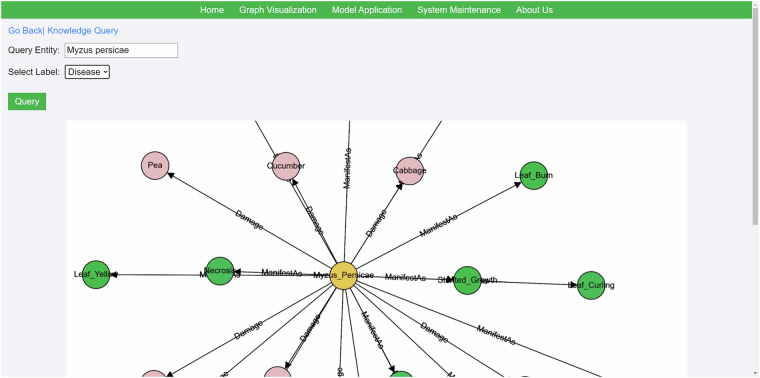
An example of knowledge querying. Users can interact with the knowledge graph by dragging and dropping to fully display and view all related information.

Knowledge service system provides users with an intuitive query interface through its user-friendly frontend. Users simply input the agricultural crop or pest-related information they wish to query and initiate the search by clicking the query button. At this point, the user’s query request is passed to the backend system. The backend system first identifies and confirms the entity name input by the user, and then utilizes the powerful querying capabilities of the Neo4j graph database to construct precise query statements using Cypher language. During the Neo4j querying process, the system conducts rapid searches within the graph database based on the entity name, exploring along paths of nodes and relationships to identify all information relevant to the queried entity. This process includes not only directly related data but also potentially includes indirectly connected entities and attributes through complex relationship networks. Neo4j’s efficient indexing and graph traversal algorithms ensure the speed and accuracy of queries, enabling users to quickly obtain comprehensive and precise query results. Due to CropDP-KG’s reliance on the powerful capabilities of the Neo4j graph database and the precise control offered by the Cypher query language, the accuracy of its query results is nearly impeccable. Cypher language is specifically designed for graph databases, allowing for declarative expressions of complex graph traversals and pattern matching, thereby ensuring the precision and reliability of queries. Each query matches nodes and relationships within the graph database precisely, guaranteeing that the returned data aligns perfectly with user requirements. Once the query is completed, the system integrates the collected data and returns it to the user, typically presented in a graphical format. This allows users to easily visualize detailed information about the queried entity and its relationships with other entities. This graph database-based querying method not only enhances query efficiency but also strengthens users’ exploration and understanding capabilities of the knowledge graph. Overall, CropDP-KG’s approach leverages the capabilities of Neo4j to empower users with efficient and insightful exploration of agricultural knowledge through advanced graph database technology. Figure 6 shows a query example focusing on the specific entity “peach aphid”. In this example, using the query functionality of CropDP-KG knowledge graph, the user successfully retrieves detailed information and data related to “peach aphid (Myzus persicae)”.

#### Knowledge question-answering

**Fig. 7:**
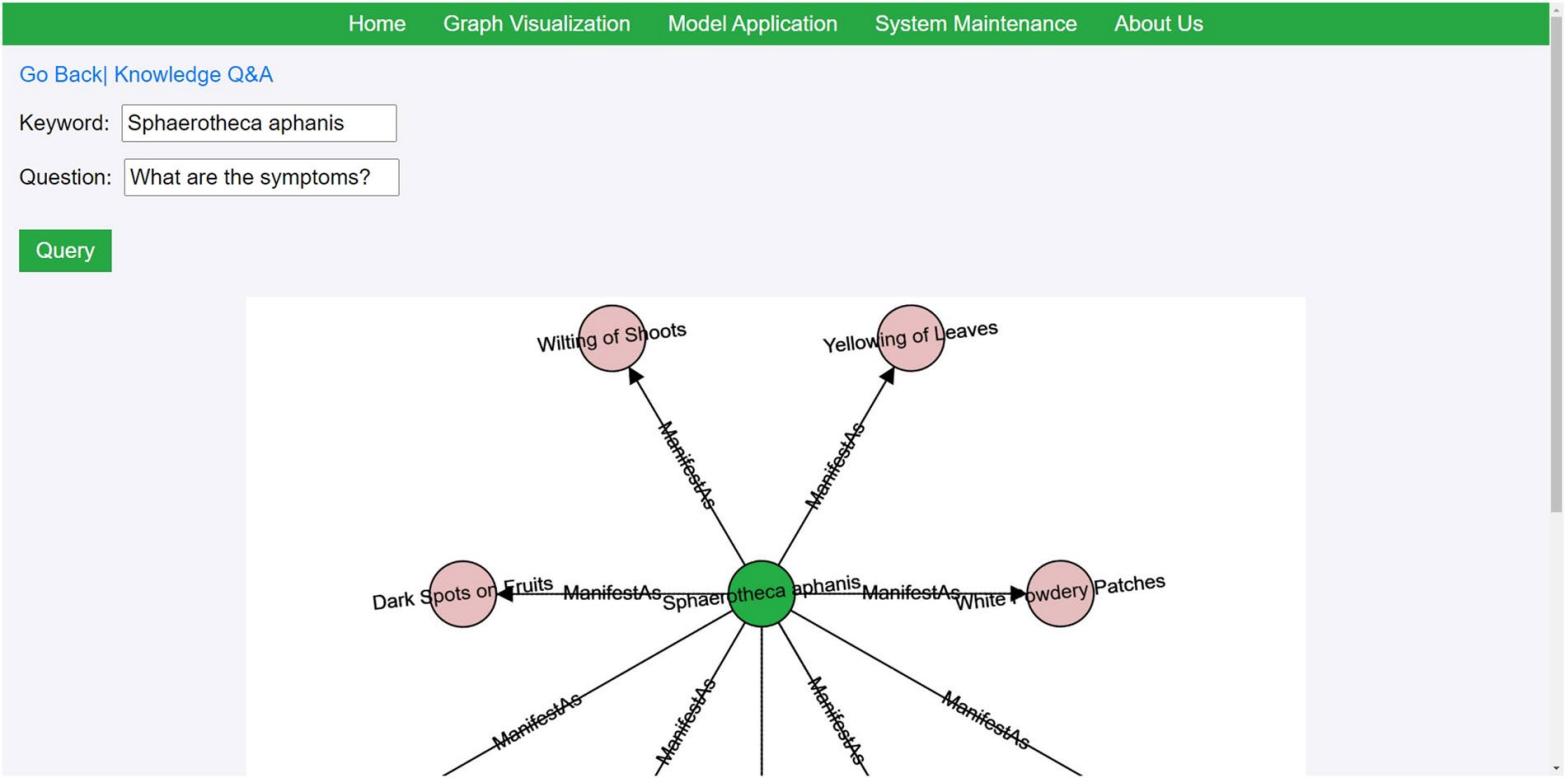
An example of a knowledge question-answering input interface.

Powered by CropDP-KG, knowledge question-answering aims to provide users with a fast and accurate way to address queries related to crop diseases and pests. Upon entering keywords and detailed questions through the frontend interface, the system’s backend initiates a series of intelligent processing steps. Initially, the system identifies entities and their relationships within the input text, then constructs precise query statements tailored to the knowledge base. Once the query statements are formulated, the system deeply searches the Neo4j graph database to retrieve the most relevant information related to the question. The retrieved answers are then carefully organized and presented in an easily understandable format to the frontend users. Users can initiate queries by entering keywords and specific questions in this interface. The keywords typically correspond to predefined entities in the knowledge graph. Since CropDP-KG is a Chinese knowledge graph, the keywords are presented in Chinese. Users can input their questions in either Chinese or English. The backend system is equipped with powerful language processing capabilities to recognize and handle queries in both languages. Figure 7 provides an example of a symptom query related to “strawberry powdery mildew (Sphaerotheca aphanis)”.

#### Knowledge management

To enable users to conveniently contribute and enhance CropDP-KG data, we have designed a user-friendly data collection page. On this page, users can easily submit their feedback, suggestions, and necessary contact information. The frontend interface is straightforward, guiding users through the input process. Once users submit their information, the backend system immediately processes and verifies the data rigorously to ensure accuracy and reliability. Processed data is securely stored in the Neo4j graph database, further enriching and expanding the knowledge graph’s content. Upon completion of storage, the system automatically sends a confirmation message to users, informing them that their submission has been successfully received and processed properly. This process not only enhances user engagement in knowledge graph development but also ensures continuous updating and optimization of knowledge graph data, making it more comprehensive and aligned with practical application needs.

## Data Records

CropDP-KG is provided in CSV format with triplet data (entity, relationship, entity), totaling 21,961 triplets. These data files have been publicly released on Figshare^36^ for use by both the academic community and the public. For a detailed description of these files, please refer to Table 3.

**Table 3:** The annotation format of six entities in unstructured data.

File name	Content
named entity.csv	Named entities obtained after annotation using Label Studio.
relationEng.csv	The triplets of pest and disease names in both Chinese and English.
relationarea.csv	The triplets of primary locations where pests and diseases occur.
relationcon.csv	The triplets of conditions for the occurrence of pests and diseases.
relationcrop.csv	The triplets of crops affected by pests and diseases.
relationpart.csv	The triplets of crop parts affected by pests and diseases.
relationsym.csv	The triplets of symptoms of pests and diseases.
relationtem.csv	The triplets of optimal temperatures for pests and diseases.

## Technical Validation

### Entity validation

We assign the task of NER to experienced domain experts who use the Labeling Studio tool to complete detailed entity annotation. To ensure the accuracy and reliability of the annotations, we organize a group of master’s and doctoral students from the School of Forestry to meticulously review the expert annotations. For any discrepancies and disputes that arise during the review process, we establish a weekly coordination mechanism, where relevant experts and graduate students meet for focused discussions. Within this framework, we aim to reach a consensus on annotation standards through in-depth communication and professional debate, ensuring high standards and consistency in the dataset annotation quality.

### Triple validation

In our research, triples serve as the core elements in constructing knowledge graphs, and their accuracy and quality are of paramount importance to us. We rigorously control the precision of the triples to ensure that they meet high standards throughout the construction process, thereby eliminating the need for additional knowledge graph linkage verification steps.

Quality management of the triples begins at the data collection stage and continues throughout the entire knowledge graph construction process. From named entity recognition to precise relationship linking, each step undergoes meticulous review and strict validation. This attention to detail and commitment to quality ensures that our triples dataset meets the required standards in terms of consistency and accuracy.

To ensure the accuracy and data quality of the triples in the knowledge graph, our research team has implemented a rigorous sampling verification mechanism. We randomly selected 5% of the triples from the dataset for in-depth and detailed verification. This task was carried out by a team of six senior professors, associate professors, and subject experts who possess extensive expertise and experience in the relevant fields. In this sampling, we reviewed a total of 1,098 triples and found only one error, which was an improper relationship annotation. We have made the necessary correction. Given that the error rate is lower than 0.1%, we believe that the impact of this error on the overall data quality is negligible, and therefore, we have decided not to give it special consideration.

During the verification process, the expert team followed a standardized review procedure, scrutinizing each triple’s subject, predicate, and object. They not only checked the accuracy of entity naming but also validated the logical consistency and reasonableness of the relationships between entities. This process significantly reduces the likelihood of annotation errors and ensures the high quality of the dataset.

The results of the verification indicate that the proportion of erroneous triples is extremely low and can be considered negligible. This outcome reflects the high level of consistency in the annotation process and confirms the high accuracy of our dataset, fully meeting our stringent standards. Through such meticulous verification, we are confident in the reliability and validity of the dataset, believing it will provide a solid foundation for subsequent scientific research and applications.

### CropDP-KG validation

CropDP-KG is built on input triples within the Neo4j graph database. To ensure the accuracy of CropDP-KG, we employed our custom-developed knowledge service system for detailed visual inspection. This system allows us to closely examine the specific content and structure of the knowledge graph, enabling a comprehensive review of the data’s accuracy and coherence. Through this visual inspection, we can scrutinize each aspect of the knowledge graph to confirm its reliability and correctness.

During the dataset construction process, we followed three core steps. First, we employed the Label Studio annotation platform to perform NER to ensure the accuracy of all entities. This process was entirely reliant on manual annotation. Although we developed an NER model to reduce future annotation costs, the data used in the subsequent triple construction was still manually annotated, rather than being derived from the model’s training data. Second, the construction of triples was manually performed by domain experts, with strict quality control throughout the process. Among a sample of 1,098 triples, only one error was identified and corrected. It can be said that all the data used to construct the knowledge graph in Neo4j underwent manual annotation and validation, with an error rate of less than 0.1%. This minor error is considered negligible. Therefore, we have strong reason to believe that the final CropDP-KG is flawless in terms of accuracy.

## Data Availability

The raw data obtained through web scraping is available at https://github.com/dadadaray/CropDP-KG/tree/CropDP-KG-originData. The CropDP-KG project is available at https://github.com/dadadaray/CropDP-KG/tree/Knowledge-System.
